# Optimal Heart Rate May Improve Systolic and Diastolic Function in Patients with Fontan Circulation

**DOI:** 10.3390/jcm12083033

**Published:** 2023-04-21

**Authors:** Keiichi Hirono, Teruhiko Imamura, Kaori Tsuboi, Shinya Takarada, Mako Okabe, Hideyuki Nakaoka, Keijiro Ibuki, Sayaka Ozawa

**Affiliations:** 1 Department of Pediatrics, Faculty of Medicine, University of Toyama, Toyama 930-0194, Japan; ktsuboi05@gmail.com (K.T.);; 2 Second Internal Medicine, Faculty of Medicine, University of Toyama, Toyama 930-0194, Japan

**Keywords:** heart rate, Fontan, heart failure, overlap length

## Abstract

(1) Background: The optimal heart rate, at which the E-wave and A-wave stand adjacent without any overlaps in the Doppler transmitral flow echocardiography, is associated with maximum cardiac output and favorable clinical outcomes in adult patients with systolic heart failure. However, the clinical implication of the echocardiographic overlap length in patients with Fontan circulation remains unknown. We investigated the relationship between heart rate (HR) and hemodynamics in Fontan surgery patients with and without beta-blockers. (2) Methods and Results: A total of 26 patients (median age 1.8 years, 13 males) were enrolled. At baseline, the plasma N-terminal pro-B-type natriuretic peptide was 2439 ± 3483 pg/mL, the fraction area change was 33.5 ± 11.4%, the cardiac index was 3.55 ± 0.90 L/min/m^2^, and the overlap length was 45.2 ± 59.0 msec. Overlap length was importantly decreased after the one-year follow-up (7.60 ± 78.57 msec, *p* = 0.0069). Positive correlations were noted between the overlap length and A-wave and E/A ratio (*p* = 0.0021 and *p* = 0.0046, respectively). Ventricular end-diastolic pressure was significantly correlated with the overlap length in non-beta-blocker patients (*p* = 0.0483). (3) Conclusion: Overlap length may reflect the status of ventricular dysfunction. Hemodynamic preservation at lower HR could be critical for cardiac reverse remodeling.

## 1. Introduction

Single ventricle disease refers to a group of severe cardiac conditions in which one ventricle predominates and supplies cardiac output to the entire body, necessitating staged surgical palliation such as Glenn surgery and Fontan surgery, which culminates in the Fontan circulation [1]. The Fontan surgery is the ultimate functional repair procedure to improve deoxygenation in patients with anatomic or functional single ventricles [2]. Due to advancements in surgical techniques and perioperative management, mid-term survival rates have reached satisfactory levels. Risk factors for postoperative complications after Fontan surgery include elevated pulmonary pressures, ventricular morphology, and ventricular dysfunction [3,4]. Increased cardiac filling pressures and a decreased ability of the Fontan circulation to maintain cardiac output can result from either systolic or diastolic ventricular dysfunction or excessive ventricular afterload.

Current guidelines strongly advise the administration and up-titration in beta-blockers in patients with systolic heart failure (HF) due to evidence that the drugs reduce mortality and morbidity and encourage cardiac reverse remodeling [5,6,7,8]. The results of large clinical trials suggest that lowering heart rate (HR) is critical for further lowering mortality in patients with HF with reduced ejection fraction who have relatively higher HR with sinus rhythm [9]. With decreasing HR, the clinical benefit of HR reduction appears to plateau. Thus, the target HR to maximize prognostic benefit remains controversial [10].

Pulse wave transmitral flow Doppler echocardiography may be a suitable tool to assess the relationship between HR and left ventricular filling. A Doppler echocardiography procedure to assess transmitral flow has been proposed recently to assess optimal HR in each individual with systolic HF. At sinus tachycardia, the E- and A-waves merge [11]. A merged A-wave is higher in a healthy cohort because the HR increases, probably to compensate for decreased left ventricular filling [12]; however, in HF patients with reduced atrial function, such compensation would not work. As the HR decreases, the widths of the E- and A-waves do not change and only the diastole is prolonged, which may not increase end-diastolic volume [12]. Patients’ cardiac output can be maximized when both the E-wave and A-wave in the transmitral flow echocardiography stand adjacent without any overlaps. Patients with optimal HR, as confirmed by Doppler echocardiography, may have better clinical outcomes than those with sub-optimal HR.

However, the clinical implication and prognostic impact of Doppler echocardiography-derived optimal HR among those with Fontan circulation remains unknown. This knowledge should improve the management of this cohort through risk stratification and provide us with the possibility of Doppler echocardiography-guided HR adjustment. We investigated the relationship between HR and hemodynamics in Fontan surgery patients with and without beta-blockers in this study.

## 2. Materials and Methods

### 2.1. Patients

All consecutive patients who received Glenn and/or Fontan surgery and were followed at our institute between June 2012 and July 2021 were retrospectively registered and included in this study. In summary, the Fontan procedure was carried out using the standard total cavopulmonary connection method, with a GORETEX artificial conduit placed between the inferior vena cava and the pulmonary artery. Following Fontan’s operation, all patients were followed by expert pediatricians according to the guideline-recommended standard manner.

The major exclusion criteria were: (1) patients who required cardioversion to terminate tachycardia; (2) the presence of or suspected hyperthyroidism; (3) following the implantation of a permanent pacemaker and/or implantable cardioverter defibrillator (except for patients who had temporary epicardial wire implantations during surgery); (4) atrial fibrillation with Wolff–Parkinson–White syndrome; (5) atrioventricular block (second degree or greater) or sick sinus syndrome; and (6) pheochromocytoma patients or suspects.

Informed consent was obtained from all patients or their parents according to institutional guidelines. This study protocol adheres to the ethical guidelines of the 1975 Helsinki Declaration, as evidenced by the prior approval by the University of Toyama’s Research Ethics Committee.

### 2.2. Echocardiographic Assessment

**Figure 1:**
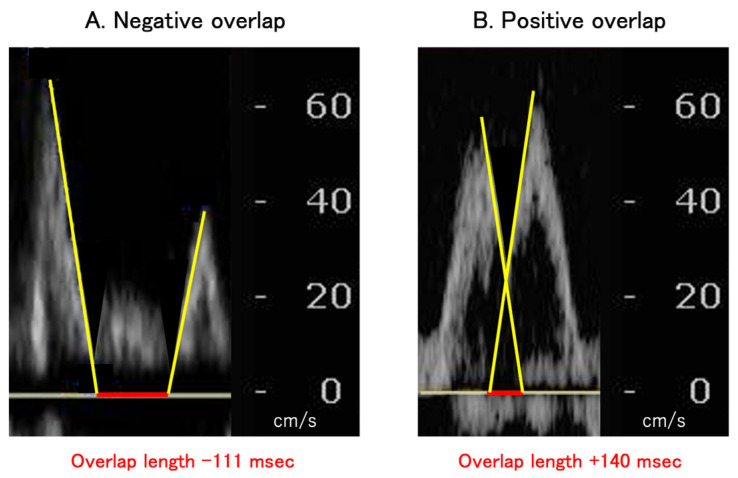
Examples of a case with negative overlap (**A**) and a case with positive overlap (**B**). In the case of A, a patient had an HR of 60 bpm and a deceleration time of 137 msec. E-wave and A-wave did not overlap. In the case of B, another patient had an HR of 131 bpm and a deceleration time of 160 msec. E-wave and A-wave considerably overlapped.

The actual HR at rest was measured at the time of index discharge for hemodynamic evaluation with echocardiogram and right heart catheterization. Expert sonographers blinded to the study’s protocol performed transthoracic echocardiography following current American Society of Echocardiography guidelines. Fraction area change (FAC) was used to evaluate ventricular systolic function. The deceleration time of the E-wave was measured using pulse Doppler echocardiography in the apical four-chamber view at trans-atrioventricular valvular flow. At the trans-atrioventricular valvular flow, the overlap between E-wave and A-wave was measured. If the two waves did not overlap, the distance between them was expressed as a negative value (Figure 1) [13].

### 2.3. Study Protocol

This is a retrospective study of patients who had Glenn and/or Fontan surgery with or without beta-blockers and had single ventricle physiology. Patients with systolic dysfunction began with beta-blockers following catheterization. The day when beta-blockers were initiated was defined as day 0. Beta-blockers were continued for at least one year. The clinical and hemodynamic status was assessed one year later. A date was compared between baseline and follow-up variables in the patients with and without beta-blockers.

### 2.4. Data Collection

Before starting beta-blockers, demographic, laboratory, medication, echocardiographic, and catheterization data were collected. Laboratory, echocardiographic, and catheterization data were obtained one year later again. Changes in hemodynamic parameters, including overlap length, were the primary endpoint. The effects of beta-blockers were counted as a secondary endpoint.

### 2.5. Statistical Analysis

Continuous variables, ordinal descriptive variables, and categorical variables were expressed as means ± SD, medians (ranges), numbers, and percentages, respectively. The unpaired *t*-test, nonparametric Mann–Whitney U test, or Kruskal–Wallis test was used to compare continuous variables, whereas categorical variables were compared employing the χ^2^ statistics or Fisher’s exact test, as appropriate. Paired *t*-tests used the parameters between baseline and follow-up. Pearson’s correlation analysis was used to examine the relationships between the same parameters before and after the Fontan operation. The intraclass correlation coefficient was used as a statistical method to assess measurement variability among observers. An intraclass correlation coefficient of ≥0.70 was considered to indicate acceptable reliability. Statistical analyses were performed using JMP software (version 16; SAS institute, Cary, NC, USA). A *p*-value < 0.05 was considered statistically significant.

## 3. Results

### 3.1. Baseline Characteristics

A total of 26 patients (median age 1.8 (0.4–3.6) years, 13 males) were included (Table 1). Twenty (76.9%) of the patients had a single right ventricle, while six (23.1%) had a single left ventricle. All patients underwent Glenn surgery at 0.8 ± 0.5 years old and 15 patients underwent Fontan surgery at 2.4 ± 0.8 years old. The plasma N-terminal pro-B-type natriuretic peptide level was 2439 ± 3483 pg/mL; the FAC level was 33.5% ± 11.4%; and the cardiac index level was 3.55 ± 0.90 L/min/m^2^. The overlap length was 45.2 ± 59.0 msec. During follow-up, 11 patients were given beta-blockers such as carvedilol and bisoprolol. There were no statistically significant differences in the demographics, laboratory, and outcomes between the two groups. Several baseline hemodynamic characteristics, including FAC, A-wave, and pulmonary vascular resistance, were significantly different between the two groups.

### 3.2. Hemodynamics between Baseline and Follow-Up Parameters

**Figure 2:**
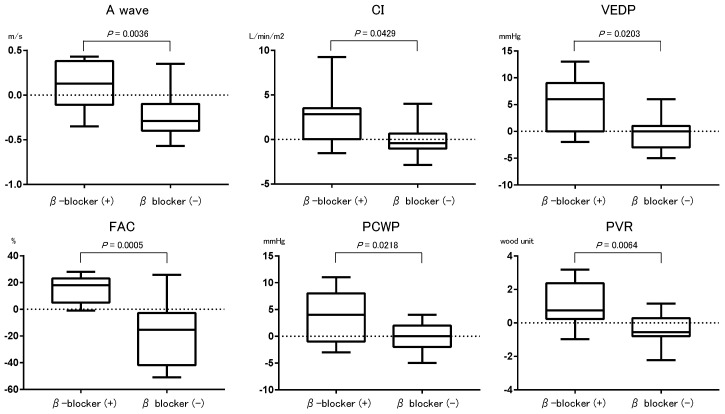
Amount of change in variables between baseline and follow-up data of patients with and without beta-blockers. The upper and lower borders of the box represent the upper and lower quartiles. The middle horizontal line represents the median. The upper and lower whiskers represent the maximum and minimum values of nonoutliers. CI, cardiac index; VEDP, ventricular end-diastolic pressure; FAC, fraction area change; PCWP, pulmonary capillary wedge pressure; PVR, pulmonary vascular resistance; β-blocker (+): beta-blocker group, β-blocker (−): non-beta-blocker group.

Table 2 shows the trends in laboratory, echocardiographic, and catheterization data from baseline (index discharge) to one year later. Overlap length was significantly reduced in all patients and beta-blockers patients (*p* < 0.05 for both, Table 2). FAC and A-wave on echocardiogram, and the cardiac index on catheterization were significantly improved in beta-blockers patients compared to those in non-beta-blocker patients, whereas pulmonary vascular resistance, pulmonary capillary wedge pressure, and ventricular end-diastolic pressure (VEDP) were not improved (*p* < 0.05 for all, Table 3 and Figure 2).

### 3.3. Relationship between Overlap Lengths and Other Variables

**Figure 3:**
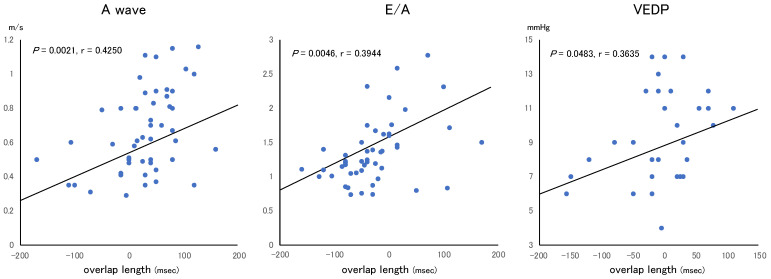
Positive correlations between overlap length and each parameter. VEDP, ventricular end-diastolic pressure.

Positive correlations were discovered between the overlap length and A-wave frequency (Figure 3). The correlation r values between the overlap length and A-wave and E/A ratio were 0.4250 (*p* = 0.0021) and 0.3944 (*p* = 0.0046), respectively. On catheterization, VEDP was significantly correlated with the overlap length in patients not taking beta-blockers (Figure 3). There were no significant correlations between the amount of change in overlap length between baseline and follow-up data and amount of change in functional parameters between baseline and follow-up data.

Intraclass correlations for echocardiographic measurements were summarized in Supplementary Materials Table S1. Overall, there was a good correlation between the two readers.

## 4. Discussion

This is the first report that overlap length was negatively correlated with A-wave, E/A, and VEDP in children who have undergone Fontan surgery. Furthermore, beta-blockers improved hemodynamic data such as systolic and diastolic function, and cardiac index in children.

### 4.1. Fontan Circulation and Ventricular Dysfunction

The afterload on the systemic ventricle is increased by Fontan surgery via cavopulmonary connection. Cardiac output is decreased in the Fontan circulation by the impaired preload [2,14]. The ventricle can easily become trapped in a vicious cycle in which low preload causes remodeling, decreased compliance, and increased filling pressures. The ventricle in the Fontan circulation results secondarily in systolic and diastolic dysfunction [15]. Thus, patients with Fontan circulation have generally subclinical HF [2,14]. In this study, higher levels of NT-pro-BNP were found, which could indicate subclinical HF. Elevated diastolic pressure decreases cardiac filling and exacerbates systemic venous hypertension, which has important implications for long-term prognosis [16]. These findings corroborated our findings that VEDP was mildly elevated. Diastolic ventricular dysfunction is more common than systolic ventricular dysfunction after Fontan surgery [17]. Echocardiographic ventricular diastolic dysfunction is common, with more than half of patients in the large Pediatric Heart Network Fontan cross-sectional study meeting diastolic dysfunction criteria [18,19]. Potential diastolic dysfunction is associated with an increased risk of adverse clinical outcomes during mid-term follow-up [17]. The lower FAC and A-wave in beta-blockers than in non-beta-blocker patients in our data could be due to diastolic dysfunction.

### 4.2. Fontan Circulation and Optimal Heart Rate

We recently proposed an equation that uses the deceleration time of the E-wave obtained by transmitral Doppler echocardiography to calculate the ideal HR for each individual with systolic dysfunction [13]. We hypothesized that when the overlap length between the E- and A-waves in the transmitral Doppler echocardiogram is zero, the cardiac output would be maximal. We tried to elucidate the optimal HR in patients with Fontan surgery. However, there was no evident relationship between FAC and cardiac index. In patients with systolic dysfunction, unstable hemodynamics with relatively low blood pressure may prevent adequate beta-blocker up-titration. The ideal HR may differ depending on the deceleration time in each individual with a specific clinical situation and each hemodynamic situation in Fontan surgery patients.

### 4.3. Fontan Circulation and Beta-Blockers

In adult clinical trials, beta-blockers have been shown to promote ventricular remodeling, lower levels of free radicals and neurohumoral toxic factors, reduce arrhythmias and thus HF symptoms, decrease mortality and morbidity, and are expected to be a significant advance in HF treatment [20,21]. Previous large clinical trials have shown that beta-blockers such as carvedilol, bisoprolol, and metoprolol succinate extended-release reduce mortality and hospitalization in patients with HF and a low ejection fraction [22,23,24]. There was no significant difference in the tolerability of bisoprolol and carvedilol at target doses in Japanese patients with HF with reduced ejection fraction [22]. Clinical efficacy and safety were comparable, though bisoprolol reduced HR, and carvedilol reduced plasma BNP more significantly.

Carvedilol is effective in pediatric patients with congenital heart disease but another study failed to confirm its clinical efficacy in children and adolescents with systolic HF [20,25,26,27]. In single ventricle patients, the addition of carvedilol to standard therapy, which included diuretics, digoxin, and ACE inhibitors, reduced HF symptoms and improved clinical parameters such as systolic dysfunction [28]. We retrospectively evaluated the clinical effects of beta-blockers in Fontan surgery patients, and hemodynamic data such as CI, VEDP, and CVP improved after treatment. Our data support that beta-blockers improve hemodynamics in patients with congenital heart disease with single ventricle morphology and may improve prognosis.

In our study, PVR in patients with beta-blockers was slightly elevated above the baseline during 1-year follow-up, and serial change in PVR was higher in patients using beta-blockers than that that in patients without beta-blockers. A large retrospective cohort study that included 568 pulmonary arterial hypertension patients who received β1-selective blockers had a similar survival rate and time to clinical worsening events compared with untreated patients [29]. Changes in pulmonary hemodynamics and right ventricular size and function measure during the 20-month follow-up were similar between patients who were treated with and without beta-blockers [30]. In a pulmonary hypertension model of rats, bisoprolol increased right ventricular contractility and filling and partially restored right ventricular-arterial coupling despite having no change in pulmonary pressure [31]. Carvedilol was able to reduce right ventricular hypertrophy and dilation [32]. Thus, the pulmonary vascular effects of beta-blockers are not fully understood and are controversial. Moreover, their effect on patients with single ventricle physiology has not been elucidated and requires further investigation.

### 4.4. Limitations

There are several limitations to this study. First and foremost, this was a single-center, retrospective study with a limited sample size. Second, there was variability in clinical and hemodynamic status. Echocardiographic imaging and catheterization data were obtained at different times and under different hemodynamic conditions, thus limiting the correlation between these two modalities. There was also inter-rater variability. Third, the clinical significance, and prognostic significance are unknown.

## 5. Conclusions

To the best of our knowledge, this is the first report suggesting that optimal HR may be associated with diastolic function in patients with Fontan circulation. Because of the lower cardiac positional energy per minute, hemodynamic preservation at low HR may be necessary for future cardiac reverse remodeling. Adequate management of HR may improve chronic HF in children undergoing Fontan surgery.

## Figures and Tables

**Table 1:** Baseline characteristics of the patients.

Patient Characteristics	All (n = 26)	β-Blocker Group (n = 11)	Non-β-Blocker Group (n = 15)	*p*-Value
Ventricular physiology				
Right ventricle	20 (76.9%)	10 (90.9%)	10 (66.7%)	0.1973
Left ventricle	6 (23.1%)	1 (9.1%)	5 (33.3%)	0.1973
Demographics				
Male	13 (50.0%)	5 (45.4%)	8 (53.3%)	1.0000
Age (years)	1.8 (0.4–3.6)	0.6 (0.3–4.1)	1.8 (0.5–2.3)	0.4816
Glenn surgery	26 (100%)	11 (100%)	15 (100%)	1.0000
Age of Glenn surgery	0.8 ± 0.5	0.9 ± 0.5	0.8 ± 0.5	0.4801
Fontan surgery	15 (57.7%)	4 (36.4%)	11 (73.3%)	0.1089
Age of Fontan surgery	2.4 ± 0.8	2.9 ± 1.0	2.2 ± 0.7	0.1733
Age at last follow-up	5.4 ± 3.9	4.7 ± 4.5	6.0 ± 3.4	0.1765
Body surface area (m^2^)	0.45 ± 0.22	0.42 ± 0.27	0.46 ± 0.17	0.2868
Heart rate (bpm)	114.0 ± 19.3	116.9 ± 22.7	111.9 ± 16.0	0.3755
Systemic blood pressure (mmHg)	90.0 ± 12.4	96.6 ± 13.9	85.1 ± 8.2	0.0453
Diastolic blood pressure (mmHg)	50.7 ± 10.0	55.2 ± 11.5	47.4 ± 7.0	0.0807
Oxygen saturation (%)	84.7 ± 6.3	85.5 ± 5.6	84.0 ± 6.6	0.2973
Symptom				
Heart failure	16 (61.54%)	9 (81.82%)	7 (46.67%)	0.1092
Arrhythmia	9 (34.62%)	7 (63.64%)	2 (13.33%)	0.0135
Dearth	1 (3.85%)	1 (9.09%)	0 (0%)	0.4231
Medication				
Beta-blocker	11 (42.3%)	11 (100%)	0 (0%)	<0.0001
Diuretic	22 (84.62%)	10 (90.91%)	12 (80.0%)	0.6137
ACE inhibitor	13 (50.0%)	5 (45.45%)	8 (53.33%)	1.0000
Anticoagulant	20 (76.92%)	11 (100%)	9 (60.0%)	0.0237
Antiplatelet	24 (92.31%)	11 (100%)	13 (86.67%)	0.4923
Pulmonary vasodilator	13 (50.0%)	5 (45.45%)	8 (53.33%)	1.0000
Antiarrhythmic agent	5 (19.23%)	3 (27.27%)	2 (13.33%)	0.6196
Chest X-ray				
Cardio-thoracic ratio (%)	56.7 ± 8.8	56.8 ± 9.1	56.5 ± 8.6	0.8350
Pulmonary congestion	9 (34.62%)	4 (36.36%)	5 (33.33%)	1.0000
Electrocardiogram				
QRS duration (msec)	98.8 ± 17.1	96.5 ± 19.9	100.4 ± 14.5	0.3109
Laboratory data				
NT-pro-BNP (pg/mL)	2439 ± 3483	4180 ± 4639	1162 ± 1184	0.1258
Echocardiographic data				
Fraction area change (%)	33.5 ± 11.4	26.9 ± 9.5	42.8 ± 6.2	0.0038
E-wave (m/s)	0.87 ± 0.26	0.86 ± 0.32	0.88 ± 0.21	0.4512
E-wave deceleration time (msec)	118.6 ± 21.6	113.4 ± 24.1	122.4 ± 19.6	0.1510
A-wave (m/s)	0.71 ± 0.26	0.57 ± 0.23	0.8 ± 0.23	0.0373
E/A	1.29±0.52	1.46±0.52	1.18±0.50	0.9033
Overlap length (msec)	45.2 ± 59.0	24.3 ± 56.4	605 ± 57.5	0.1244
Catheterization data				
Cardiac index (L/min/m^2^)	3.55 ± 0.90	3.36 ± 0.78	3.63 ± 0.93	0.4577
Pulmonary vascular resistance (wood unit)	1.33 ± 0.52	1.04 ± 0.35	1.45 ± 0.53	0.0319
Central venous pressure (mmHg)	9.8 ± 3.2	8.9 ± 3.5	10.2 ± 3.0	0.6450
Pulmonary capillary wedge pressure (mmHg)	8.9 ± 3.0	7.6 ± 2.8	9.5 ± 2.9	0.2154
Ventricular end-diastolic pressure (mmHg)	9.3 ± 3.3	8.5 ± 3.3	9.7 ± 3.2	0.5055

ACE, angiotensin-converting enzyme; BNP, brain natriuretic peptide.

**Table 2:** Comparison between baseline and follow-up variables.

	All (n = 26)			β-Blocker Group (n = 11)			Non-β-Blocker Group (n = 15)		
Patient Characteristics	Baseline	Follow-Up	*p*-Value	Baseline	Follow-Up	*p*-Value	Baseline	Follow-Up	*p*-Value
Age (years)	1.8 (0.4–3.6)	2.9 (1.4–5.0)	0.0344	0.6 (0.3–4.1)	2.5 (1.2–5.0)	0.0930	1.8 (0.5–2.3)	2.9 (1.6–3.3)	0.0535
Body surface area (m^2^)	0.43 (0.27–0.52)	0.51 (0.40–0.61)	0.0369	0.26 (0.22–0.60)	0.41 (0.33–0.67)	0.1396	0.43 (0.33–0.49)	0.53 (0.45–0.57)	0.0152
Heart rate (bpm)	114.0 ± 19.3	103.1 ± 17.2	0.0045	116.9 ± 22.7	100.73 ± 14.7	0.0078	111.9 ± 16.00	104.8 ± 18.6	0.1626
Systemic blood pressure (mmHg)	90.0 ± 12.4	92.0 ± 12.5	0.5400	96.6 ± 13.9	93.5 ± 15.1	0.6199	85.1 ± 8.2	91.0 ± 10.0.	0.1064
Diastolic blood pressure (mmHg)	50.7 ± 10.0	51.6 ± 12.4	0.7574	55.2 ± 11.5	52.9 ± 15.8	0.7108	47.4 ± 7.04	50.6 ± 8.91	0.1880
Oxygen saturation (%)	84.7 ± 6.3	89.7 ± 5.6	0.0012	85.5 ± 5.6	89.4 ± 6.1	0.1023	84.0 ± 6.6	90.0 ± 5.2	0.0061
Cardio-thoracic ratio (%)	56.7 ± 8.8	55.2 ± 9.5	0.1765	56.8 ± 9.1	56.5 ± 10.7	0.8351	56.5 ± 8.6	54.3 ± 8.4	0.1060
QRS duration (msec)	98.7 ± 17.1	108.0 ± 23.2	0.0201	96.5 ± 19.9	109.4 ± 28.2	0.0969	100.4 ± 14.5	106.9 ± 18.9	0.1202
NT-pro-BNP (pg/mL)	2439 ± 3483	977 ± 1292	0.0152	4180 ± 4639	1614 ± 1642	0.066	1162 ± 1184	510.3 ± 634.2	0.0138
Fraction area change (%)	33.5 ± 11.4	40.3 ± 7.1	0.0558	26.9 ± 9.5	41.7 ± 5.0	0.0006	42.8 ± 6.2	39.2 ± 8.1	0.6330
E-wave (m/s)	0.87 ± 0.26	0.88 ± 0.27	0.8554	0.86 ± 0.32	0.96 ± 0.33	0.3397	0.88 ± 0.21	0.83 ± 0.19	0.3487
E-wave deceleration time (msec)	118.6 ± 21.6	133.7 ± 28.0	0.0214	113.4 ± 24.1	133.8 ± 35.4	0.0773	122.4 ± 19.6	133.7 ± 22.5	0.1640
A-wave (m/s)	0.71 ± 0.26	0.61 ± 0.20	0.1014	0.57 ± 0.23	0.68 ± 0.20	0.2592	0.8 ± 0.23	0.57 ± 0.19	0.0017
E/A	1.29 ± 0.52	1.49 ± 0.50	0.1715	1.46 ± 0.52	1.32 ± 0.27	0.477	1.18 ± 0.50	1.60 ± 0.59	0.0446
Overlap length (msec)	45.2 ± 59.0	7.6 ± 78.6	0.0069	24.3 ± 56.4	−5.2 ± 60.4	0.0086	60.5 ± 57.8	17.0 ± 88.4	0.1235
Cardiac index (L/min/m^2^)	3.55 ± 0.9	3.77 ± 1.64	0.9852	3.36 ± 0.78	4.18 ± 2.08	0.488	3.63 ± 0.93	3.47 ± 1.12	0.6489
Pulmonary vascular resistance (wood unit)	1.33 ± 0.52	1.31 ± 0.79	0.5406	1.04 ± 0.35	1.73 ± 0.86	0.2804	1.45 ± 0.53	1.01 ± 0.56	0.0622
Central venous pressure (mmHg)	9.8 ± 3.2	10.8 ± 2.2	0.1035	8.9 ± 3.5	10.4 ± 2.4	0.1476	10.2 ± 3.0	11.0 ± 2.0	0.3868
Pulmonary capillary wedge pressure (mmHg)	8.9 ± 3.0	9.4 ± 2.2	0.4181	7.6 ± 2.8	9.7 ± 2.5	0.1723	9.5 ± 2.9	9.1 ± 2.0	0.6702
Ventricular end-diastolic pressure (mmHg)	9.3 ± 3.3	9.8 ± 2.2	0.5753	8.5 ± 3.3	10.6 ± 2.2	0.2607	9.7 ± 3.2	9.2 ± 2.0	0.5708

BNP, brain natriuretic peptide.

**Table 3:** Amount of change in variables between baseline and follow-up data.

Change (Follow-Up—Baseline)	All (n = 26)	β-Blocker Group (n = 11)	Non-β-Blocker Group (n = 15)	*p*-Value
Δheart rate (bpm)	−10.9 ± 17.5	−16.2 ± 16.2	−7.1 ± 18.6	0.2869
Δsystemic blood pressure (mmHg)	2.1 ± 16.7	−3.2 ± 20.6	5.9 ± 13.3	0.0771
Δdiastolic blood pressure (mmHg)	0.9 ± 14.2	−2.3 ± 19.8	3.2 ± 9.0	0.0540
Δoxygen saturation (%)	5.1 ± 6.9	3.8 ± 7.0	6.0 ± 7.2	0.3234
Δcardio-thoracic ratio (%)	−1.4 ± 5.1	−0.4 ± 5.6	−2.2 ± 4.9	0.6204
ΔQRS duration (msec)	9.2 ± 18.5	12.8 ± 23.2	6.5 ± 15.3	0.4303
ΔNT-pro-BNP (pg/mL)	−1462 ± 2805	−2566 ± 4124	−652 ± 897	0.5506
Δfraction area change (%)	−2.7 ± 23.6	14.9 ± 9.9	−15.6 ± 23.3	0.0005
ΔE-wave (m/s)	0.010 ± 0.26	0.095 ± 0.32	−0.053 ± 0.21	0.2323
ΔE-wave deceleration time (msec)	15.2 ± 31.5	20.5 ± 34.5	11.3 ± 29.7	0.7633
ΔA-wave (m/s)	−0.100 ± 0.29	0.095 ± 0.26	−0.24 ± 0.24	0.0036
ΔE/A	0.19 ± 0.79	−0.12 ± 0.63	0.42 ± 0.83	0.0822
Δoverlap length (msec)	−37.5 ± 88.8	−50.5 ± 116.9	−19.6 ± 28.2	0.6588
Δcardiac index (L/min/m^2^)	1.04 ± 2.57	2.35 ± 2.95	0.075 ± 1.93	0.0429
Δpulmonary vascular resistance (wood unit)	2.29 ± 1.23	1.16 ± 1.25	−0.35 ± 0.83	0.0064
Δcentral venous pressure (mmHg)	2.1 ± 4.0	3.8 ± 4.4	0.8 ± 3.5	0.0900
Δpulmonary capillary wedge pressure (mmHg)	1.5 ± 4.2	4.0 ± 4.7	−0.3 ± 2.9	0.0218
Δventricular end-diastolic pressure (mmHg)	1.9 ± 4.9	5.0 ± 5.5	−0.5 ± 3.1	0.0203

BNP, brain natriuretic peptide.

## Data Availability

The authors confirm that the data supporting the findings of this study are available within the article.

## Supplementary Materials

The following supporting information can be downloaded at: https://www.mdpi.com/article/10.3390/jcm12083033/s1, Table S1: Inter-rater reliability for echocardiographic measurements.
