# Supplementary material for: Optimal Heart Rate May Improve Systolic and Diastolic Function in Patients with Fontan Circulation
Source: J Clin Med. 2023 Apr 21;12(8):3033. doi: 10.3390/jcm12083033 (PMC10146582; doi:10.3390/jcm12083033)
Supplement: Supplementary file 1 [file jcm-12-03033-s001.zip › jcm-2304631-supplementary.pdf]

Supplemental Table S1. Inter-rater reliability for echocardiographic measurements

Echocardiographic measurements	Correlation coefficients
Baseline	
Fraction area change	0.8554
E-wave	0.9640
E-wave deceleration time	0.7147
A-wave	0.9547
Overlap length	0.9116
Follow-up	
Fraction area change	0.7173
E-wave	0.9918
E-wave deceleration time	0.9520
A-wave	0.8374
Overlap length	0.9325
